# An engineered glove to follow finger function in rheumatoid arthritis: an observational prospective study

**DOI:** 10.1007/s00296-023-05444-w

**Published:** 2023-09-13

**Authors:** A. Sulli, P. Clini, G. Bruzzone, A. Signori, T. Vojinovic, S. Paolino, E. Gotelli, E. Hysa, V. Smith, M. Cutolo

**Affiliations:** 1 Laboratory of Experimental Rheumatology and Academic Division of Clinical Rheumatology, Department of Internal Medicine, University of Genova, Genoa, Italy; 2 IRCCS San Martino Polyclinic Hospital, Genoa, Italy; 3 Geriatric Clinic, Department of Internal Medicine, IRCCS San Martino Polyclinic Hospital, University of Genova, Genoa, Italy; 4 Department of Health Sciences (DISSAL), Section of Biostatistics, University of Genova, Genoa, Italy; 5 Department of Internal Medicine, Ghent University, Ghent, Belgium; 6 Department of Rheumatology, Ghent University Hospital, Ghent, Belgium; 7 Unit for Molecular Immunology and Inflammation, Inflammation Research Center (IRC), Vlaams Instituut Voor Biotechnologie (VIB), Ghent, Belgium

**Keywords:** Rheumatoid arthritis, Engineered glove, Functional performance, Hand disability, Clinimetric indexes

## Abstract

The engineered Hand Test System (HTS) glove has shown high reliability in assessing the baseline functional status of rheumatoid arthritis (RA) hand. Starting from this achievement, the aim of the present observational prospective study was to assess the functionality of the single fingers of rheumatoid hand at follow-up. Eighty RA patients performed HTS glove tests at baseline and among these fifty-six patients were re-tested after 7 months. The HTS glove parameters [Touch Duration (TD), Movement Rate (MR), Inter Tapping Interval (ITI)] were correlated with disease activity and disability clinimetric indexes [Disease Activity Score 28 joint count—C-reactive protein (DAS28-CRP), Clinical Disease Activity Index (CDAI), Simplified Disease Activity Index (SDAI), Health Assessment Questionnaire—Disability Index (HAQ-DI), grip strength, visual analogue scale of pain (VAS), patient global assessment (PGA)], and with laboratory values. HTS glove parameters (TD, ITI, and MR) showed statistically significant correlations with clinimetric and clinical indexes at both time points (*p* < 0.05). During follow-up, a statistically significant variation of all HTS glove parameters for the fingers that have performed both the worst or best HTS test at baseline was detected (*p* < 0.05), while the mean HTS glove parameter values by considering all fingers did not show a statistically significant variation over time, as well as the traditional clinimetric indexes. Besides the objective role in assessing the RA hand function by integrating the traditional clinimetric indexes, the HTS glove seems a useful tool for evaluating worst or best finger function during time by measuring the movement speed.

## Introduction

Rheumatoid arthritis (RA) is a chronic, autoimmune, systemic inflammatory disorder that primarily affects the diarthrodial joints and the subchondral bone. The articular inflammatory process begins generally from the synovial tissue and small joints of hands are frequently involved, slowing fine finger movements [1].

The disease is characterized by the continuous alternation of two phases: the phase of disease activity, which requires a more aggressive therapy aimed at extinguishing the immune-mediated inflammation, and a remission phase, in which the disease is under control with a basic therapy. Over time, if the disease is not kept under control, RA has a chronic disabling evolution, frequently associated with systemic multi-organ manifestations with a substantial societal impact in terms of social cost, disability, and loss of productivity [1–4].

Therefore, it is important to have effective tools to obtain outcome measures as precise as possible, to assess the effectiveness of the ongoing treatment.

Currently, the most used disease activity scores to frame the patient condition are the Disease Activity Score 28 joint count—C-reactive protein (DAS28-CRP), Simplified Disease Activity Index (SDAI), and Clinical Disease Activity Index (CDAI) [5–8]. These scores provide some assessments of functional outcomes, based on clinical and biochemical signs of inflammation. As a limit, they may be affected by an operator-dependent bias in the clinical evaluation. In addition, some disease-specific questionnaires, i.e. the Health Assessment Questionnaire—Disease Index (HAQ-DI), are available to evaluate patient subjective perception of the disease and disability [9–13].

Recently, different types of engineered gloves have been developed and tested to evaluate different functional parameters of the hand joints providing quantitative data [14]. Data gloves, that use a combination of static and dynamic sensors, are able to provide parametric data that can be used in the clinical setting to evaluate hand function [15, 16]. In clinical practice, several researchers tried to demonstrate the effectiveness of data gloves in monitoring degenerative or inflammatory diseases, indicating them as valid tools for hand function assessment [17–21].

In the field of rheumatology, data gloves are currently used for research purposes only and have not yet become part of clinical practice. The hand test system (HTS) glove is a medical device that has been recently tested on RA patients [22]. This glove has shown a good sensitivity in detecting the dexterity of the finger opposition movements, and the correlation between finger functions and health status was demonstrated in RA patients [22].

The objective of this observational prospective study was to test the HTS glove in RA patients to assess the single finger function in a more detailed manner at baseline and during the follow-up and to compare the glove parameters with the scores provided by traditional RA clinical indexes.

## Methods

### Patients

Eighty consecutive adult RA patients (71 women and 9 men, mean age 62 years, mean disease duration 14 years) were enrolled at the rheumatologic outpatient clinic from January 2020 to May 2022. RA was diagnosed according to the 2010 ACR/EULAR criteria [23]. Among these, 56 RA patients (51 women and 5 men, mean age 61 years, mean disease duration 13 years) were followed-up for a second clinical assessment, regardless of the undergoing treatment. Due to COVID pandemic, several patients were lost during follow-up.

Eligible RA patients for the study were those who did not have permanent anatomical alterations due to other morbid states, including osteoarthritis with Bouchard’s or Heberden’s nodules, carpal tunnel syndrome or tendon nodules. Patients who had cognitive (Alzheimer, senile dementia, etc.) or functional (fibromyalgia, Parkinson’s) dysfunctions were also excluded from the study. These exclusion criteria were applied to exclude that the dysfunction detected by the glove was due to a condition different from RA.

The drug taken by patients were not considered neither at entry nor during follow-up, as the aim of the study was to evaluate the ability of the glove to objectively quantify the changes of finger function in RA patient, regardless of the medications taken. Also the disease activity status of patient at baseline was not part of inclusion or exclusion criteria, as the aim of the study was to analyse possible changes of finger function even in stable or remission disease activity.

The hand function was tested at baseline in 80 RA patients and re-tested in 56 of them, after a mean follow-up of 7 ± 2 months. At each evaluation, patients performed HTS glove test and clinical and laboratory parameters were recorded, including disease activity scales, HAQ-DI questionnaire, visual analogue scale of pain (VAS), and grip strength assessment (see below) [24–26].

### HTS glove test

The functional evaluation of the hand was assessed by the HTS glove, which provides a parametric assessment of the finger movements, assessing the maximal finger velocity. The glove is easy to wear and adaptable to different hand sizes. On the tip of each finger of the glove there are conductive materials, which constitute the only sensors present in this glove. The absence of additional sensors on the joints or palm guarantees that the mobility of the hand is not altered in any way by the device.

In accordance with the rules for the containment of SARS-COV-2 infection, patients were required to sanitize their hands and wear a disposable non-sterile vinyl glove before wearing the HTS glove.

The measurements were carried out by the same operator (PC). All patients were tested in the afternoon, between 2 and 4 pm, to minimize the interference due to circadian rhythms of the night time inflammatory reaction and the related joint morning stiffness. [27–29].

During the exercises both the operator and the patient remained silent, and the room had no open windows or other sources of visual or noise distraction.

The HTS glove was tested on both the dominant and non-dominant hand in each RA patient.

**Fig. 1:**
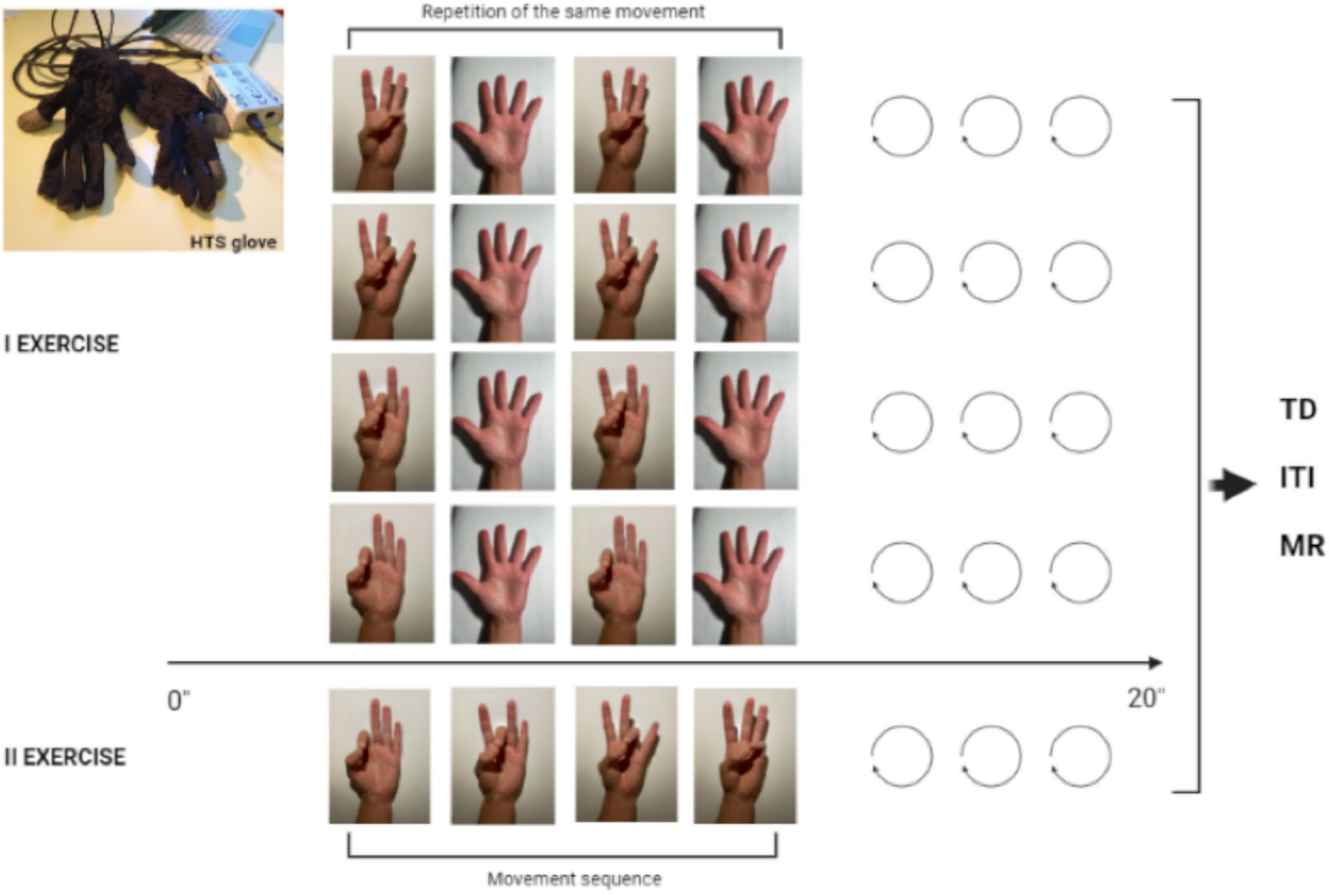
Representation of the two exercises that patients were asked to perform with HTS glove, an intensive exercise (first exercise) and a sequential one (second exercise). The first exercise consisted of having the patient repeat the same movement touching the thumb and one finger at a time as many times as possible in 20 s (in particular touching between the thumb and index finger, thumb and middle finger, thumb and ring finger, and thumb and little finger). The second exercise involved the sequential and repeated opposition of each finger with the thumb for 20 s, to perform as many touches as possible. TD, touch duration; ITI, inter tapping interval; MR, movement rate

Patients were given two types of exercises, an intensive exercise and a sequential one (Fig. 1).

The first exercise consisted of having the patient repeat a sequence of touch as many times as possible in 20 s, involving the thumb and one finger at a time. In particular, the sequence consisted of touching between the thumb and index finger, thumb and middle finger, thumb and ring finger, and thumb and little finger.

The second exercise involved the sequential and repeated opposition of each finger with the thumb for 20 s, to perform as many touches as possible. Patients were asked to perform the movements by maximally expanding the extension of the joint at the end of each individual touch. Patients were allowed to try these movements several times before recording the test to become familiar with the exercise and avoid errors due to misunderstanding. The beginning and end of each exercise were dictated by the operator.

The software was able to recognize only the correctly completed sequences, eliminating the wrong or incomplete ones, which did not result in the final count.

The parametric data recorded by the HTS glove were acquired through a data acquisition card (USB-1208FS, Measurement Computing, USA) and analysed by the HTS software. The three quantitative data analysed were as follow: (1) Touch Duration (TD): indicates the average time of contact between the fingers during the sequence, measured in milliseconds (ms); (2) Movement Rate (MR): indicates the frequency of the touches, quantified in hertz (Hz); (3) Inter Tapping Interval (ITI): average time between one touch and the other, objectified in milliseconds.

During follow-up patients did not repeat neither the first nor the second HTS glove exercise, to prevent training in HTS glove test possibly interfering with the results.

### Disease activity and hand strength

RA disease activity was assessed by DAS28-CRP, CDAI, and SDAI [2]. Patient condition was evaluated by VAS of pain and patient global assessments (PGA) [30–34]. The Italian version of the HAQ-DI questionnaire was used for the assessment of RA disability [12].

Furthermore, the number of tender joints (NTJ), number of swollen joints (NSJ), morning stiffness (MS), C-reactive protein (CRP), and erythrocyte sedimentation rate (ESR) were also assessed at any visit.

An analogic dynamometer *(Smedley Dynamometer, Gima, Gessate, Italy)* was used to assess hand grip strength [24], and the measurements were conducted in accordance with Mathiowetz’s guidelines [35].

### Ethical approval

HTS glove investigation was approved by the Regional Ethical Committee of San Martino Polyclinic Hospital (ID 12822-661, May, 8th 2023) and every patient involved in the study provided the informed consent to enter the study and manage their clinical data. The whole study was conducted in accordance with the principles of the Declaration of Helsinki and good clinical practice.

### Statistical analysis

A minimum sample size of 49 patients was needed to find as significant a correlation between glove parameters and clinical data of 0.40 with a statistical power of 80% and a level of significance of 5%.

Data were reported as mean with standard deviation or median with interquartile range (IQR) for skewed data. To assess the change between first and second measurement, the non-parametric Wilcoxon test for skewed data and paired samples *t* test for the other data were used.

For the comparison between baseline and follow-up of HTS glove data, we conducted a repeated measure ANOVA to examine the potential impact of age, gender, and disease duration.

The Spearman’s correlation coefficient was used to correlate glove parameters with clinical data. *p* values < 0.05 were considered statistically significant and were adjusted for multiple comparisons using the false-discovery rate (fdr) approach. Stata software (v.17; StataCorp) was used for the computation.

We did not use a normality test that, for small sample sizes, have low statistical power to detect problems with normality of data, and we checked all distributions graphically. Furthermore, there is no established standard for interpreting correlation levels, as numerous arbitrary thresholds exist, and the contextual relevance of the outcomes must be taken into account. Typically, correlations below 0.4 are classified as weak [36]. Given the lack of universally recognized thresholds, we have chosen not to assign labels indicating the strength of the coefficients.

## Results

Clinical parameters of RA patients are reported in Table 1.

**Table 1:** Clinical features of RA patients enrolled in the study: 80 patients were assessed only at baseline and 56 patients were re-tested after a mean follow-up of 7 ± 2 months

Parameters	Baseline	Follow-up
	80 RA patients	56 RA patients
Demographic data		
Mean age ± SD (years)	62 ± 14	61 ± 13
Sex (F/M)	71/9	51/5
Mean disease duration ± SD (years)	14 ± 9	13 ± 8
Dominant hand		
Right-handed # (%)	74 (92.5)	50 (89.3)
Left-handed # (%)	3 (3.75)	3 (5.35)
Ambidextrous # (%)	3 (3.75)	3 (5.35)
Auto-antibody profile		
Positive ACPA # (%)	59 (73.75)	39 (69)
Positive RF # (%)	50 (62.5)	33 (59)
Double positivity RF + ACPA # (%)	42 (52.5)	27 (48)
Double negativity RF + ACPA # (%)	15 (18.75)	14 (25)
DAS 28-CRP		
Remission (DAS28-CRP < 2.6) # (%)	41 (51.25)	31 (55)
Low disease activity (2.6 < DAS28-CRP < 3.2) # (%)	9 (11.25)	6 (11)
Moderate disease activity (3.2 < DAS28-CRP < 5.1) # (%)	24 (30)	16 (28)
High disease activity (DAS28-CRP > 5.1) # (%)	4 (5)	2 (3)
CDAI		
Remission (≤ 2.8) # (%)	24 (30)	11 (20)
Low disease activity (≤ 10) # (%)	30 (37.5)	32 (57)
Moderate disease activity (10 < CDAI ≤ 22) # (%)	18 (22.5)	9 (16)
High disease activity (> 22) # (%)	6 (7.5)	3 (5)
SDAI		
Remission (≤ 3.3) # (%)	24 (30)	13 (23)
Low disease activity (3.3 < SDAI ≤ 11) # (%)	26 (32.5)	28 (50)
Moderate disease activity (11 < SDAI ≤ 26) # (%)	23 (29)	11 (20)
High disease activity (> 26) # (%)	5 (6)	2 (4)

*RF* rheumatoid factor, *ACPA* anti-citrullinated protein autoantibodies, *DAS28-CRP* Disease Activity Score 28 joint count—C-reactive protein, *CDAI* Clinical Disease Activity Index, *SDAI* Simplified Disease Activity Index, *SD* standard deviation

Clinical activity scale scores, laboratory values, and HTS glove parameters at baseline and during follow-up are described in Table 2. As reported, no statistically significant variation of clinical parameters was identified between first and second assessments.

**Table 2:** Clinical activity scale scores, laboratory values and HTS glove parameters of the 56 RA patients at baseline and after follow-up. HTS glove values represent the mean of all fingers from both hands. Statistical significance (*p*) of the comparison between the two assessments is also reported

	Baseline 56 RA patients	Follow-up 56 RA patients	*p* value
Traditional assessment			
DAS28-CRP Mean ± SD, (Median) [IQR]	2.87 ± 1.31 (2.5) [1.83–4.0]	2.53 ± 1.31 (2.20) [1.63–2.92]	0.29
HAQ-DI Mean ± SD, (Median) [IQR]	13 ± 13.55 (7.5) [7.5–21.25]	10.5 ± 12.27 (6) [1–14]	0.55
Grip strength Mean ± SD pounds, (Median) [IQR]	44.9 ± 16.77 (45) [30–55]	49.35 ± 14.42 (50) [40–60]	0.14
CDAI Mean ± SD, (Median) [IQR]	8.36 ± 8.63 (5.37) [2.3–14.1]	7.69 ± 7.34 (5) [3–9]	0.31
SDAI Mean ± SD, (Median) [IQR]	9.56 ± 10.62 (4.75) [2–12.75]	8.54 ± 7.57 (5.43) [3.30–9.77]	0.66
NTJ Mean ± SD, (Median) [IQR]	2.68 ± 4.9 (0) [0–3.25]	2.18 ± 3.02 (0) [0–1]	0.71
NSJ Mean ± SD, (Median) [IQR]	0.96 ± 1.8 (0) [0–1]	0.76 ± 1.81 (0) [0–0]	0.99
VAS of pain Mean ± SD, (Median) [IQR]	3.29 ± 2.49 (3.36) [1–5]	3.41 ± 2.34 (3) [2–475]	0.23
PGA Mean ± SD, (Median) [IQR]	1.26 ± 2.51 (1.31) [0–0.75]	3.41 ± 2.34 (4) [2–5]	0.32
MS Mean ± SD minutes, (Median) [IQR]	20.8 ± 38.1 (0) [0–30]	14.5 ± 23.12 (0) [0–10]	0.99
ESR Mean ± SD, (Median) [IQR]	32.62 ± 25.28 (30) [10–50.25]	28.77 ± 21.56 (27) [9–45]	0.75
CRP mg/L Mean ± SD, (Median) [IQR]	7.9 ± 8.08 (4) [2–11]	6.61 ± 9.54 (3.10) [2–8.4]	0.78
HTS glove parameters			
MR Mean ± SD Hertz, (Median) [IQR]	2.10 ± 0.94 (2) [1.32–2.72]	2.13 ± 0.93 (2.12) [1.31–2.95]	0.22
ITI Mean ± SD msec, (Median) [IQR]	345.62 ± 220.7 266.9 [181.6–416.7]	328 ± 204.6 (270.2) [178.7–424.3]	0.36
TD Mean ± SD msec, (Median) [IQR]	273.45 ± 158.15 (228.9) [150.38–331.3]	272.67 ± 151 (232.7) [158.4–337.0]	0.23

*DAS28-CRP* Disease Activity Score 28 joint count—C-reactive protein, *HAQ-DI* Health Assessment Questioner Disability Index, *CDAI* Clinical Disease Activity Index, *SDAI* Simplified Disease Activity Index, *VAS* visual analogue scale, *PGA* patient global assessment, *CRP* C-reactive protein, *MS* morning stiffness, *min* minutes, *ESR* erythrocyte sedimentation rate, *NTJ* number of tender joints, *NSJ* number of swollen joints, *TD* touch duration (msec), *MR* movement rate (hertz), *ITI* inter tapping interval (msec), *SD* standard deviation, *IQR* interquartile range

The correlations between HTS glove parameters (mean of all fingers from both hands) and disease clinical indexes (DAS28-CRP, CDAI, SDAI), scores of subjective and objective disability (VAS, PGA, HAQ-DI, Grip strength), laboratory values (CRP, ESR), and other clinical parameters (morning stiffness, NTJ, NSJ) are reported in Table 3. In particular, statistically significant correlations were observed between HTS glove parameters and DAS28-CRP, HAQ-DI, CDAI, SDAI, VAS, PGA, and Grip strength values at first assessment (80 patients evaluated). At second assessment, statistically significant correlations were again observed between HTS glove parameters and DAS28-CRP, HAQ-DI, CDAI, SDAI, VAS, and grip strength values (56 patients evaluated). Inconstant or absence of clinically significant correlations were found between HTS glove parameters and PGA, MS, CRP, ESR, NTJ, and NSJ at the follow-up (see Table 3 for statistical significances and Spearman’s *r* values).

**Table 3:** Correlation between HTS glove parameters and other clinical parameters at baseline (80 patients assessed) and after follow-up (56 patients re-tested) in our cohort of RA patient. HTS glove value obtained were the mean of all fingers from both hands

	Baseline 80 RA patients	Follow-up 56 RA patients		Baseline 80 RA patients	Follow-up 56 RA patients
	DAS28-CRP			Grip strength	
TD	*r* = 0.26 *p* = 0.039	*r* = 0.29 *p* = 0.036	TD	*r* = − 0.35 *p* = 0.009	*r* = − 0.39 *p* = 0.004
MR	*r* = − 0.32 *p* = 0.010	*r* = − 0.27 *p* = 0.047	MR	*r* = 0.34 *p* = 0.009	*r* = 0.39 *p* = 0.005
ITI	*r* = 0.36 *p* = 0.006	*r* = 0.26 *p* = 0.091	ITI	*r* = − 0.26 *p* = 0.042	*r* = − 0.29 *p* = 0.031
	HAQ-DI			CRP	
TD	*r* = 0.38 *p* = 0.004	*r* = 0.35 *p* = 0.011	TD	*r* = 0.20 *p* = 0.11	*r* = 0.18 *p* = 0.21
MR	*r* = − 0.43 *p* < 0.001	*r* = − 0.35 *p* = 0.011	MR	*r* = − 0.10 *p* = 0.40	*r* = − 0.13 *p* = 0.38
ITI	*r* = 0.43 *p* < 0.001	*r* = 0.28 *p* = 0.040	ITI	*r* = 0.08 *p* = 0.53	*r* = 0.09 *p* = 0.55
	CDAI			ESR	
TD	*r* = 0.33 *p* = 0.009	*r* = 0.28 *p* = 0.042	TD	*r* = 0.37 *p* = 0.033	*r* = 0.28 *p* = 0.047
MR	*r* = -0.37 *p* = 0.004	*r* = − 0.27 *p* = 0.047	MR	*r* = − 0.27 *p* = 0.042	*r* = − 0.27 *p* = 0.048
ITI	*r* = 0.35 *p* = 0.006	*r* = 0.24 *p* = 0.091	ITI	*r* = 0.16 *p* = 0.20	*r* = 0.24 *p* = 0.13
	SDAI			MS	
TD	*r* = 0.26 *p* = 0.033	*r* = 0.33 *p* = 0.017	TD	*r* = 0.12 *p* = 0.731	*r* = 0.04 *p* = 0.77
MR	*r* = − 0.32 *p* = 0.010	*r* = − 0.33 *p* = 0.016	MR	*r* = − 0.06 *p* = 0.20	*r* = 0.11 *p* = 0.47
ITI	*r* = 0.34 *p* = 0.008	*r* = 0.26 *p* = 0.091	ITI	*r* = 0.28 *p* = 0.021	*r* = 0.08 *p* = 0.59
	VAS			NTJ	
TD	*r* = 0.40 *p* < 0.001	*r* = 0.31 *p* = 0.023	TD	*r* = 0.21 *p* = 0.076	*r* = 0.26 *p* = 0.091
MR	*r* = − 0.40 *p* < 0.001	*r* = − 0.31 *p* = 0.023	MR	*r* = − 0.10 *p* = 0.40	*r* = 0.04 *p* = 0.77
ITI	*r* = 0.31 *p* = 0.011	*r* = 0.29 *p* = 0.037	ITI	*r* = 0.39 *p* = 0.004	*r* = 0.21 *p* = 0.17
	PGA			NSJ	
TD	*r* = 0.46 *p* < 0.001	*r* = 0.33 *p* = 0.017	TD	*r* = 0.21 *p* = 0.059	*r* = 0.28 *p* = 0.040
MR	*r* = − 0.44 *p* < 0.001	*r* = − 0.26 *p* = 0.087	MR	*r* = 0.07 *p* = 0.54	*r* = 0.19 *p* = 0.20
ITI	*r* = 0.37 *p* = 0.004	*r* = 0.21 *p* = 0.091	ITI	*r* = 0.30 *p* = 0.013	*r* = 0.18 *p* = 0.21

*r* Spearman’s *r*, *p p* value, *DAS28-CRP *Disease Activity Score 28 joint count—C-reactive protein, *HAQ-DI *Health Assessment Questioner Disability Index, *CDAI *Clinical Disease Activity Index, *SDAI *Simplified Disease Activity Index, *VAS *visual analogue scale, *PGA *patient global assessment, *CRP *C-reactive protein, *MS *morning stiffness, *min *minutes, *ESR *erythrocyte sedimentation rate, *NTJ *number of tender joints, *NSJ* number of swollen joints, *TD *touch duration (msec), *MR *movement rate (hertz), *ITI *inter tapping interval (msec)

*p* values were adjusted for multiple comparisons using the false-discovery rate approach

The variation (delta) of the mean values between the two assessments of both HTS glove parameters (of whole fingers) and clinical parameters was also calculated. However, the correlations between the variations were found not statistically significant, as expected due to the lack of statistically significant changes of clinical parameters between first and second assessment and the non-consensual variance (see Table 4 for further information).

**Table 4:** The table reports the correlations between the variation (delta) of HTS glove parameters (mean of all fingers of the two hands) and variations (delta) of RA clinimetric indexes during follow-up in 56 patients

	Δ DAS28-CRP		Δ Grip strength		Δ VAS		Δ HAQ-DI	
	*r*	*p*	*r*	*p*	*r*	*p*	*r*	*p*
Δ TD	0.10	0.463	− 0.26	0.651	0.23	0.624	0.06	0.673
Δ ITI	0.12	0.381	− 0.27	0.237	0.17	0.225	− 0.15	0.264
Δ MR	0.01	0.966	0.24	0.092	− 0.12	0.394	0.11	0.423
Δ worst finger TD	0.16	0.229	− 0.11	0.427	0.21	0.138	− 0.04	0.776
Δ worst finger ITI	0.14	0.293	− 0.08	0.565	0.14	0.315	− 0.23	0.086
Δ worst finger MR	0.07	0.581	0.10	0.464	− 0.07	0.630	0.19	0.179
Δ best finger TD	0.04	0.745	− 0.01	0.921	0.15	0.299	0.11	0.435
Δ best finger ITI	0.01	0.907	− 0.42	0.421	0.25	0.080	− 0.12	0.392
Δ best finger MR	0.02	0.843	0.11	0.427	− 0.26	0.061	0.02	0.905

Also the correlations between the variations (delta) of each HTS glove parameter of the worst or the best baseline finger performance (selected among all the fingers of the two hands) and RA clinimetric parameters are reported (56 patients)

*Δ DAS28-CRP* variation of DAS28-CRP between two measurement, ***Δ**** TD* touch duration variation between two measurements, ***Δ**** ITI* inter tapping interval variation between two measurements, ***Δ**** MR*: movement rate variation between two measurements, ***Δ**** worst-TD* touch duration variation between two measurements of the most affected finger, ***Δ**** worst-ITI* inter tapping interval variation between two measurements of the most affected finger, ***Δ**** worst-MR* movement rate variation between two measurements of the most affected finger, ***Δ**** best-TD* touch duration variation between two measurements of the less affected finger, ***Δ**** best-ITI* inter tapping interval variation between two measurements of the less affected finger, ***Δ**** best-MR* movement rate variation between two measurements of the less affected finger, *r* = Spearman’s *r*, *p p* value

The analysis of the mean HTS glove parameter values for each single finger (index, medium, ring, and little), assessed also for right and left hand individually, did not reveal any statistically significant correlation between the two measurements at baseline and after follow-up, as shown in Table 5.

**Table 5:** Comparison of mean HTS glove parameter values of each single finger (index, medium, ring, little) between baseline and follow-up, in 56 RA patients analysed for selected right and left hand

			Baseline 56 patients	Follow-up 56 patients	*p*
Right hand					
TD (ms)	Index	Mean ± SD (Median) [IQR]	235.95 ± 159.61 (197.55) [165.77]	274.51 ± 154.33 (239.26) [207.32]	0.99
	Medium	Mean ± SD (Median) [IQR]	260.18 ± 201.30 (203.62) [171.45]	249.23 ± 155.32 (214.16) [168.37]	0.99
	Ring	Mean ± SD (Median) [IQR]	254.90 ± 173.25 (208.99) [166.55]	262.81 ± 158.95 (212.53) [183.88]	0.99
	Little	Mean ± SD (Median) [IQR]	280.87 ± 207.64 (297.37) [187.70]	263.07 ± 158.13 (200.73) [187.70]	0.99
MR (Hz)	Index	Mean ± SD (Median) [IQR]	2.18 ± 1.00 (1.99) [1.21]	2.15 ± 1.03 (1.78) [1.66]	0.89
	Medium	Mean ± SD (Median) [IQR]	2.15 ± 1.10 (1.78) [1.19]	2.19 ± 1.01 (1.87) [1.60]	0.89
	Ring	Mean ± SD (Median) [IQR]	2.28 ± 1.20 (2.14) [1.83]	2.23 ± 1.04 (2.16) [1.37]	0.99
	Little	Mean ± SD (Median) [IQR]	2.32 ± 1.59 (2.16) [1.37]	2.06 ± 0.99 (1.94) [1.61]	0.50
ITI (ms)	Index	Mean ± SD (Median) [IQR]	331.01 ± 189.67 (291.64) [221.94]	336.58 ± 272.51 (240.97) [236.85]	0.88
	Medium	Mean ± SD (Median) [IQR]	359.37 ± 250.92 (281.51) [231.21]	327.74 ± 206.48 (261.83) [236.00]	0.88
	Ring	Mean ± SD (Median) [IQR]	306.91 ± 211.28 (218.65) [221.70]	312.19 ± 214.01 (235.73) [276.50]	0.88
	Little	Mean ± SD (Median) [IQR]	326.60 ± 229.95 (247.50) [222.03]	361.34 ± 276.75 (252.71) [276.84]	0.88
Left hand					
TD (ms)	Index	Mean ± SD (Median) [IQR]	258.67 ± 148.53 (243.29) [170.19]	285.27 ± 182.38 (242.60) [193.60]	0.39
	Medium	Mean ± SD (Median) [IQR]	248.76 ± 151.27 (216.23) [144.57]	256.24 ± 145.71 (216.91) [138.22]	0.99
	Ring	Mean ± SD (Median) [IQR]	270.58 ± 164.00 (222.38) [150.77]	278.84 ± 158.48 (230.81) [170.29]	0.99
	Little	Mean ± SD (Median) [IQR]	287.68 ± 183.18 (230.90) [224.02]	284.96 ± 161.06 (233.12) [193.82]	0.99
MR (Hz)	Index	Mean ± SD (Median) [IQR]	2.20 ± 1.01 (2.09) [1.26]	2.12 ± 0.97 (1.87) [1.77]	0.88
	Medium	Mean ± SD (Median) [IQR]	2.26 ± 1.06 (2.16) [1.25]	2.25 ± 0.98 (2.26) [1.80]	0.88
	Ring	Mean ± SD (Median) [IQR]	2.17 ± 1.00 (1.95) [1.52]	2.24 ± 1.01 (2.39) [1.65]	0.88
	Little	Mean ± SD (Median) [IQR]	2.08 ± 1.00 (1.99) [1.27]	1.98 ± 0.86 (1.89) [1.34]	0.88
ITI (ms)	Index	Mean ± SD (Median) [IQR]	321.97 ± 226.57 (256.34) [194.74]	314.32 ± 177.22 (267.89) [237.46]	0.88
	Medium	Mean ± SD (Median) [IQR]	302.33 ± 201.92 (224.82) [182.08]	295.52 ± 172.38 (221.24) [254.46]	0.88
	Ring	Mean ± SD (Median) [IQR]	311.72 ± 195.71 (240.37) [242.55]	296.06 ± 220.02 (218.70) [269.86]	0.99
	Little	Mean ± SD (Median) [IQR]	306.66 ± 214.00 (227.27) [241.12]	326.12 ± 185.66 (284.52) [279.56]	0.50
Finger performance at baseline					
TD (ms)	Worst	Mean ± SD (Median) [IQR]	346.35 ± 230.70 (281.87) [286.18]	280,16 ± 158,51 (230.66) [183.87]	**0.0232**
	Best	Mean ± SD (Median) [IQR]	189,81 ± 135,96 (149,94) [132,54]	247,93 ± 144,31 (204,92) [152,56]	**0.0083**
MR (Hz)	Worst	Mean ± SD (Median) [IQR]	1.7 ± 0.91 (1.68) [1.10]	2.18 ± 1.02 (2.20) [1.91]	**0.049**
	Best	Mean ± SD (Median) [IQR]	2.70 ± 1.20 (2.53) [1.67]	2.23 ± 1.05 (2.04) [1.77]	**0.0083**
ITI (ms)	Worst	Mean ± SD (Median) [IQR]	450.05 ± 288.21 (342,80) [299.49]	339.09 ± 250.07 (278.99) [250.44]	**0.0083**
	Best	Mean ± SD (Median) [IQR]	232.79 ± 159.67 (188.83) [128.26]	291.11 ± 174.53 (223.00) [248.68]	**0.0083**

The table also reports the comparison of mean HTS glove parameter values between baseline and follow-up, after selection for each glove parameter of the worst or the best finger performance at baseline among all the fingers of the two hands. *p* values were adjusted for multiple comparisons using the false-discovery rate approach

Significant values are in bold

*TD* touch duration, *MR* movement rate, *ITI* inter tapping interval, *SD* standard deviation, *IQR* interquartile range, *ms* milliseconds, *Hz* hertz, *p* *p* value

Therefore, we selected for each glove parameter the mean values of the worst and the best baseline finger performance (selected among all the fingers of the two hands in each patient) and analysed their variation from baseline to follow-up. This was done in the hypothesis of being able to observe over time greater clinical variations of finger function and consequently of the HTS glove parameters.

This time, we observed statistically significant changes of these HTS glove values after the follow-up (see Table 5 for statistical significances). In particular, we observed increased values of MR and reduction of TD and ITI values after follow-up of worst baseline finger performance cohort. Conversely, we detected a reduction of MR and an increment of TD and ITI after follow-up of the best baseline finger performance group.

On the other hand, by analysing the correlations between the variation (delta) of each glove parameter value and the variation of each clinical parameter value after follow-up in this subgroup of HTS glove data, no statistical significance was observed (Table 4).

This last result is of great relevance demonstrating the superior usefulness of HTS glove in detecting single finger function variations during the follow-up, to either the whole hand function or the traditional clinical evaluation scales.

Of note, no statistically significant correlations were found between HTS glove parameters and both laboratory values (ESR and CRP) and MS (Table 3). Laboratory values are non-specific parameters, being influenced by multiple factors, as well as infections, trauma, inflammatory processes different from arthritis, and the fluctuation of these parameters can manifest non-consensually to the fine variations of the parameters of the data glove.

Age, gender, and disease duration were found to have no significant impact on HTS glove values. Therefore, no adjustment for these variables was performed.

## Discussion

In a previous study, the HTS data glove demonstrated a good ability in differentiating RA patient hand function from healthy population, even in a condition of disease remission [22]. In the present study, we evaluated over time in RA patients the correlations between the variation of the HTS glove parameters, by considering the mean of all fingers, and the variation of the clinimetric indexes, but no statistically significant correlation was identified. Furthermore, no statistically significant change over time was observed for both glove parameters and clinimetric indexes.

In particular, we selected the DAS28-CRP, CDAI, SDAI as cornerstones for the evaluation of RA clinical activity, HAQ-DI, VAS, PGA, grip strength as parameters of RA clinical disability, and laboratory values, such as CRP and ESR. As assessed by DAS28-CRP and other clinical indexes, almost 60% of enrolled RA patients were in clinical remission or low disease activity, possibly explaining the above reported lack of statistical significances during the follow-up in our cohort of patients.

Subsequently, by analysing separately the right and left hand performance by HTS glove tests, considering the mean of all fingers, no statistically significant change was observed after the follow-up, most likely since not all the fingers were affected by the same intensity of the inflammatory process and consequent clinical involvement.

Afterwards, as the mean finger performance might mask the worsening or the improvement of the single finger function, we identified in each patient the single finger that had performed worse or better at baseline on the basis of each HTS glove parameter, following the change of the finger involvement severity (low TD and ITI and high MR values represent a better finger function, while high TD and ITI and low MR values denote a worst finger function).

This time, a strong statistically significant variation of all single parameters of the HTS glove was observed at follow-up, whereas the correlations between the mean HTS glove parameter changes and the clinical/clinimetric indexes were still non statistically significant.

Therefore, the study suggests that the HTS glove tests can assess over time the improvement or worsening of a finger function in selected patients, while the clinimetric indexes provide a more general view of the disease status that do not represent the fine hand function, similarly to what has been shown previously in the field of multiple sclerosis assessment with the same engineered glove [15].

Of note, the HTS glove analysis of single fingers may allow to identify and quantify the specific finger status in RA patients even in presence normal clinimetric index scores [37].

This study has some limitations. First, patient heterogeneity was large and the small cohort of enrolled patients did not allow to split the sample into subgroups according to peculiar clinical characteristics. The recruitment of a larger number of RA patients might change the statistical significance of several comparisons and correlations here tested.

Disease activity at baseline was not part of the inclusion criteria of this work. Therefore, patients were not enrolled at the time of treatment modification due to high/moderate disease activity. With hindsight, the substantial disease stability during the follow-up was a limitation, as shown by the absence of significant variations of the clinimetric indexes. This affected the assessment of hand function and the relationship between HTS glove parameters and the patient clinical condition.

However, in a previous study, the HTS glove demonstrated a good ability in differentiating RA patient hand function from healthy population, even in a condition of disease remission [22, 38].

Possibly, a longer follow-up might show larger variations in disease activity, allowing more statistically significant correlations and comparisons between the analysed parameters, together with possible links with the assessment of hands imaging [39, 40]. Also, the possibility to enroll a more homogeneous group of patients possibly divided into subgroups with moderate/high disease activity at baseline before starting a systemic treatment, or contrariwise a group sharing remission or low disease activity at enrolment. As our goal was to evaluate the ability of the glove to objectively quantify the patient RA single finger function status, regardless of the medications taken, different treatments were not considered in this study.

Several engineered gloves may include the assessment of range of motion among the parameters provided [14]. The accuracy of data interpretation and analysis (in particular concerning MR and ITI) might further be improved by the acquisition of the finger range of motion (ROM), through the presence of further specific sensor nets inside the HTS glove [21]. Even if the presence of range of motion measurements might improve the completeness of finger function assessments, the data recorded by our HTS glove are enough reliable to assess finger function in RA patients.

Of relevance, the choice of selecting and following the fingers that showed the worst and best HTS glove test performance (for each glove parameter: TD, MR, ITI), driven by the fact that globally the mean HTS glove parameter values of fingers did not differ during follow-up due to patient clinical stability, was a further successful analysis to identify the ability of HTS glove to detect fine finger dexterity changes over time.

Lastly, the associations between finger status and its swelling and tenderness was not assessed in this study. However, the difference in finger status/performance between swollen, tender, and normal fingers will be matter of further investigation.

## Conclusions

The HTS glove is confirmed to offer an objective quantification of the RA hand function and in particular the single finger status at baseline and during the follow-up by integrating the traditional clinimetric indexes.

## Data Availability

V.S. is a senior clinical investigator of the Research Foundation Flanders, Belgium (FWO; 1.8.029.20 N). The FWO was not involved in the study design, collection, analysis, and interpretation of data, writing of the report or in the decision to submit the manuscript for publication.
